# Effects of Two Natural Bisbenzylisoquinolines, Curine and Guattegaumerine, Extracted from *Isolona hexaloba* on Rhodamine Efflux by Abcb1b from Rat Glycocholic-Acid-Resistant Hepatocarcinoma Cells

**DOI:** 10.3390/molecules27093030

**Published:** 2022-05-09

**Authors:** Jacques-Aurélien Sergent, Hilarion Mathouet, Christian Hulen, Pedro Lameiras, Marc Feuilloley, Abdelhakim Elomri, Nour-Eddine Lomri

**Affiliations:** 1 Department of Biology, UFR Sciences and Techniques, University of Cergy-Pontoise, 2 Ave A. Chauvin, 95302 Cergy-Pontoise, France; sergent.jacques@gmail.com; 2 UNIROUEN, INSA Rouen, CNRS, COBRA (UMR 6014), Normandie University, 76000 Rouen, France; hmathouet@yahoo.fr (H.M.); pedro.lameiras@univ-reims.fr (P.L.); 3 Bacterial Communication and Antimicrobial Strategies Research Unit, University of Rouen Normandy, 55 rue Saint Germain, 2700 Evreux, France; hulen.marie@orange.fr (C.H.); marc.feuilloley@univ-rouen.fr (M.F.)

**Keywords:** alkaloids, curine, guattegaumerine, verapamil, efflux pump inhibition, MDR1

## Abstract

To develop new therapeutic molecules, it is essential to understand the biological effects and targets of clinically relevant compounds. In this article, we describe the extraction and characterization of two alkaloids from the roots of *Isolona hexaloba*—curine and guattegaumerine. The effect of these alkaloids on the multidrug efflux pump ABCB1 (MDR1/P-Glycoprotein) and their antiproliferative properties were studied. Compared to verapamil, a widely used inhibitor of P-gp, curine and guattegaumerine were found to be weak inhibitors of MDR1/P-Glycoprotein. The highest inhibition of efflux produced by verapamil disappeared in the presence of curine or guattegaumerine as competitors, and the most pronounced effect was achieved with curine. Altogether, this work has provided new insights into the biological effects of these alkaloids on the rat Mdr1b P-gp efflux mechanism and would be beneficial in the design of potent P-gp inhibitors.

## 1. Introduction

Plants are an extraordinary reservoir of molecules that exhibit various biological activities in many different organisms. *Isolona* (Annonaceae), a subfamily of Monodoroidae [1], is a genus consisting of about twenty-five species confined to tropical Africa and Madagascar. These plants are described [2] as trees or bushes. The molecules presented here were extracted from *Isolona hexaloba* Engl and Diels, one of the seven species growing in the humid forest of central Africa (Gabon). This plant is a 10–40 m high tree with a 60 cm diameter trunk on the ground. The bark is soft and approximately 1 cm thick. *I. hexaloba* is characterized by the horizontal positions of its petals and the ovoidal to sub-globular fruits with bumps and longitudinal ribs. The leaves are 6–30 cm long, 3–10 cm large, and sub-coriaceous [2].

Some species are used as herbal remedies in traditional medicine. *Isolona campanulata* is used as an aphrodisiac on the Ivory Coast [3]. In Zaïre, a bark decoction administered per ounce is described in local medicine as a purgative and smoked as a myorelaxant [4]. Some *Isolona* species have been chemically studied, and several natural alkaloids of these plants have shown antimalarial and anti-trypanosomal properties [5,6,7]. However, the mechanisms of action and safety of these extracts have rarely been investigated [8].

Chemoresistance is frequently encountered in the treatment of cancer and infectious diseases, and finding solutions to this problem is one of the targets of the World Health Organization. In cancer cells, as well as in infectious bacteria, chemoresistance is often related to the over-expression of efflux pumps that expel unrelated drugs or antibiotics before they can reach their targets. Namely, the resistance of cancer cells to anti-cancer agents is based on the expulsion of these compounds through a transport protein belonging to the ABC family [9]. It is well known, for instance, that alkaloids are expelled from human cells by the membrane-associated P-glycoprotein (P-gp) encoded by the MDR1 gene (ABCB1) [10].

Continuing our investigation on the medical potential of West African plants [11,12], here, we present the purification of curine and guattegaumerine—two alkaloids isolated from the roots of *Isolona hexaloba*. This family of molecules is known for its in vitro cytotoxicity, as well as its antiplasmodial and amoebicidal activities [13,14]. Curine is also characterized as a molecule with calcium pump inhibitory activity and a vasodilatory alkaloid [15]. Moreover, curine is capable of inhibiting contractions elicited by high extracellular potassium concentrations in the rat aorta and reducing the rise in the intracellular Ca2^+^ concentration in vascular smooth A7r5 cells [16]. It has also been shown that curine induces G1 arrest and cell death in hepatocarcinoma HepG2 cells [17]. More recently, Ribeiro-Filho et al. [18] demonstrated that curine inhibited the secretion of cytokines (tumor necrosis factor (TNFα), interleukine (IL)-1β, IL-6), as well as the production of nitric oxide (NO) in lipopolysaccharide (LPS), and it activated macrophages, possibly by negatively modulating Ca^2+^ influx in the same order of magnitude as verapamil.

The pharmacological activity of guattegaumerine has not yet been well established, although it was suggested to have antimitotic activity 30 years ago [19]. Additionally, more recently, Lü et al. [20] showed that guattegaumerine suppressed the increase in intracellular calcium concentration stimulated by H_2_O_2_ in a Ca^2+^-free solution on rat primary-cultured cortical neurons. Guattegaumerine seems to possess potential protection against oxidative stress injury.

Therefore, we tested the effects of curine (A), guattegaumerine (B), and semi-synthetic triacetylguattegaumerine (C) on the efflux of rhodamine 123, a specific fluorescent substrate of P-gp (MDR1) [21,22], and compared the activity of these alkaloids to that of a non-competitive inhibitor of rhodamine efflux by P-gp, or verapamil [23,24,25]. This analysis was performed by using poorly differentiated HTC rat hepatoma cells specially adapted for resistance to permeable bile acid ester (HTC-R cells) [26] and overexpressing several plasma membrane proteins representing novel members of the ABC/Mdr family, including Mdr1b.

## 2. Results

### 2.1. Evaluation of the Toxicity and Response of HTC-R Cells to Curine and Guattegaumerine Exposure

**Figure 1:**
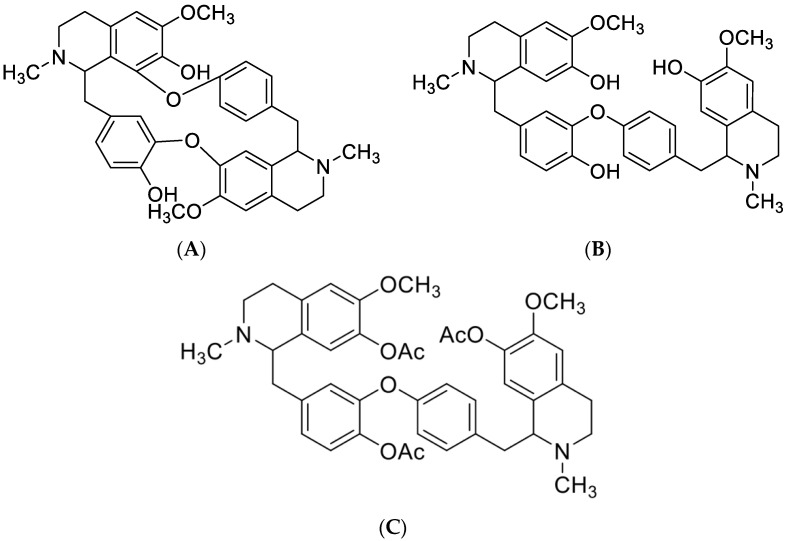
Chemical structures of compounds isolated from the roots of *Isolona hexaloba:* curine (**A**), guattegaumerine (**B**), and triacetylguattegaumerine (**C**).

In this study, we show the isolation and the characterization of two alkaloids of biological importance from the roots of *Isolona hexaloba*: curine (Figure 1A) and guattegaumerine (Figure 1B). Their structures (Figure 1) were identified through analysis of physical and spectral data (see Section 4). To determine the structure of guattegaumerine, it was necessary to perform acetylation of the free hydroxyl groups to make the hemi-synthesis of triacetylguattegaumerine (Figure 1C). Finally, spectral analysis of triacetylguattegaumerine enabled us to find the actual structure of guattegaumerine (Figure 1B).

#### 2.1.1. Structures of Compounds Isolated from *Isolona hexaloba*

**Figure 2:**
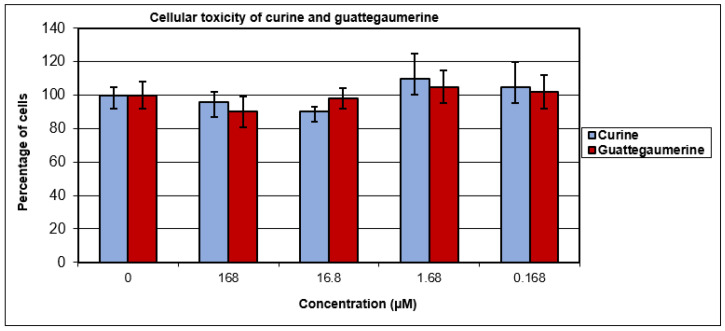
Cellular toxicity of curine and guattegaumerine. HTC-R cells were grown to confluence in 24-well plates and exposed for 24 h at the concentrations of curine or guattegaumerine indicated in abscissas. The percentage of surviving cells is plotted in ordinates. Experiments were performed three times in triplicate. Values represent the mean percentage of surviving cells ± standard deviation.

In reference to activities previously described in the literature for these two molecules [13,14,19], the cellular toxicity of curine and guattegaumerine was initially tested on confluent rat hepatocarcinoma cells (HTC-R) at concentrations ranging from 0.168 to 168 µM. The cells were exposed to the drug for 24 h. The survival of cells was measured by staining with crystal violet and compared with that of untreated cells. The percentage of living cells after exposure to drugs remained identical to that in the control (Figure 2). Moreover, no significant toxicity was observed for curine and guattegaumerine, even after 48 h of exposure.

**Figure 3:**
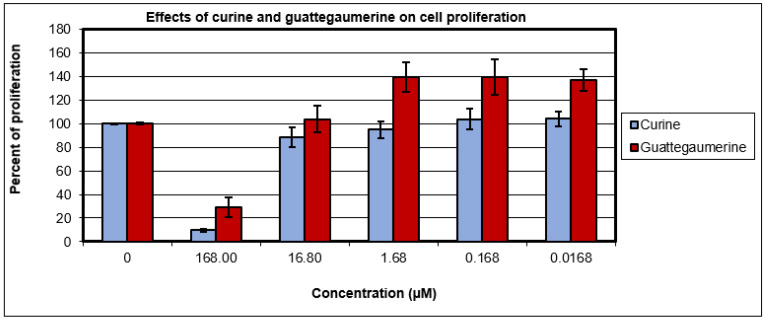
Effects of curine and guattegaumerine on the proliferation of HTC-R cells. The cells were grown in 24-well plates in the absence (control) or presence of concentrations of the curine and guattegaumerine indicated in abscissas. After 36 h of incubation, the percentage of living cells was calculated. Experiments were carried out three times in triplicate. Values represent the mean percentage of living cells ± standard deviation.

The effects of curine and guattegaumerine on the proliferation of HTC-R cells were evaluated after 36 h of growth. Figure 3 shows that 168 μM of curine or guattegaumerine inhibited cell proliferation by 90% or 70%, respectively. At 16.8 µM of curine or guattegaumerine, the proliferative activity of HTC-R cells was not significantly affected. Therefore, these two molecules appear to be nontoxic for confluent cells, but have inhibitory activity on cell proliferation at concentrations above 50 µM.

#### 2.1.2. Effects of Curine and Guattegaumerine on Efflux Pumps

One feature of cancer cells is their ability to acquire resistance to anticancer agents. One of the cellular mechanisms involved in anticancer drug resistance is the expulsion of drugs through membrane proteins, such as the P-glycoprotein or P-gp (permeability glycoprotein) encoded by the MDR1 gene. Conventionally, P-gp is an efflux protein belonging to the ABC transporter family [27]. Overexpression of P-gp efflux proteins provides HTC-R cells with increased resistance to anticancer drugs, since these toxins are rapidly expelled from the cells [26]. The purpose of our test was, then, to evaluate the effect of curine and guattegaumerine on the P-gp efflux pump, since a blockage of this pump could optimize the activity of anticancer molecules. The method was based on the fluorescent properties of rhodamine 123, a molecule that is specifically carried out of the cells by this efflux pump [21]. The effect of curine and guattegaumerine was compared to that of verapamil, a reference inhibitor of the P-gp-dependent efflux system [24]. On the basis of the results presented in Figure 2 and according to the literature on verapamil [24], the drugs were used at a concentration of 100 µM on confluent cells.

**Figure 4:**
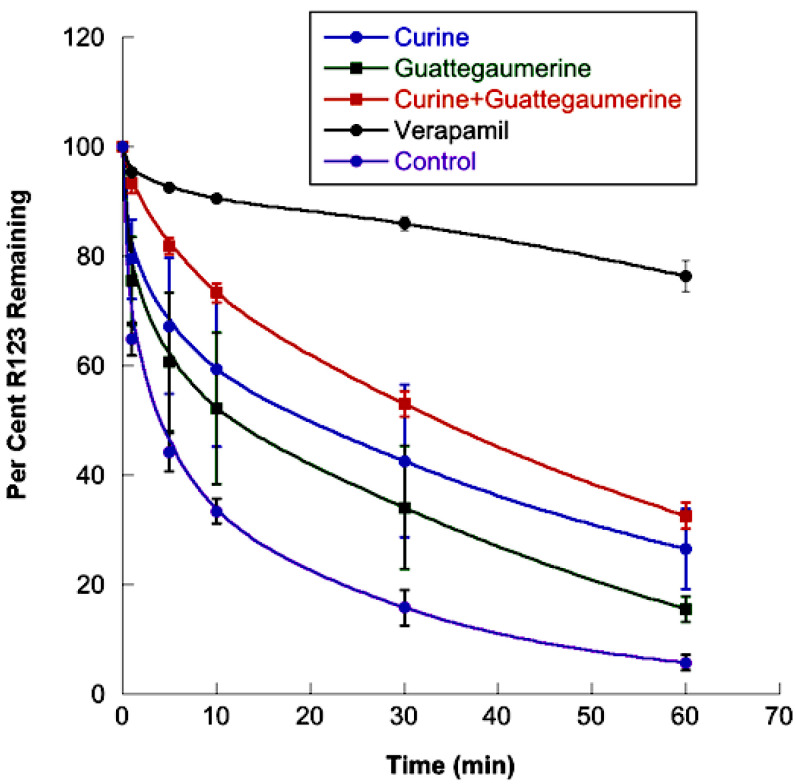
Effects of curine and guattegaumerine on the efflux of rhodamine from HTC-R cells. The cells were grown to confluence in DMEM, and rhodamine accumulated for 90 min at 37 °C. After washing the cells, the efflux of rhodamine was initiated at 37 °C by the addition of PBS (control) or PBS containing curine or guattegaumerine at 100 μM. For the time indicated in abscissa, the amount of rhodamine output from the cells was measured by fluorescence. The results are expressed as a percentage of rhodamine 123 remaining in the cells. These results are representative of at least two independent experiments and show the mean ± SD of triplicate plates.

The results presented in Figure 4 show that, after accumulation in HTC-R cells, rhodamine 123 was actively transported out of the cells by the P-gp protein for 10 min, and then diffused more slowly for at least 60 min. In the presence of verapamil, we observed a decrease of 80% in the efflux process. In the presence of guattegaumerine or curine, the initial rate of rhodamine efflux decreased by 22% or 41%, respectively. When curine and guattegaumerine were added together, the efflux of rhodamine was reduced by 65%. This percentage of inhibition could be interpreted as the sum of the individual effects of curine (41%) and guattegaumerine (22%), suggesting the existence of an additive process of inhibition. We also tested triacetylguattegaumerine (100 μM). This alkaloid did not inhibit the release of rhodamine from the cells (results identical to the control), suggesting that the addition of acetyl groups significantly reduced the effect of guattegaumerine on rhodamine efflux.

**Figure 5:**
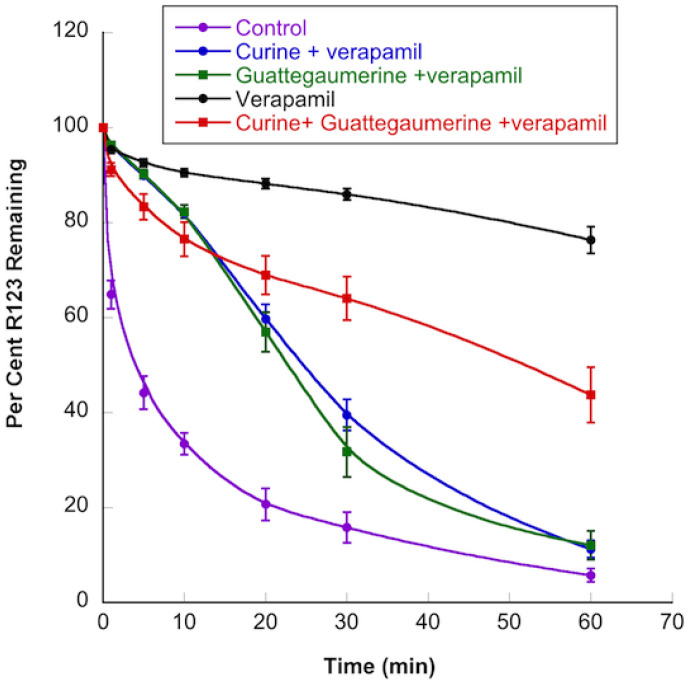
Effects of curine and guattegaumerine on the inhibition of rhodamine 123 efflux by verapamil. HTC-R cells were grown to confluence in DMEM and rhodamine accumulated for 90 min at 37 °C. After washing the cells, the efflux of rhodamine was initiated at 37 °C by the addition of PBS (control) or PBS containing verapamil, verapamil and curine, or verapamil and guattegaumerine at 100 μM each. For the time indicated in abscissa, the amount of rhodamine output from cells was measured by fluorescence. The results are expressed as a percentage of rhodamine 123 remaining in the cells. They are representative of at least two independent experiments and show the mean ± SD of triplicate plates.

When the efflux of rhodamine was measured in the presence of both verapamil and curine or guattegaumerine (Figure 5), we observed that the inhibitory activity of verapamil was suppressed by curine or guattegaumerine. In the presence of verapamil, the average speed of rhodamine output from cells increased four-fold when curine or guattegaumerine was added. When curine and guattegaumerine were added together with verapamil (Figure 5), the level of rhodamine efflux inhibition was identical to that observed with curine plus guattegaumerine without verapamil (see Figure 4 for comparison). The inhibitory effect of verapamil was completely blocked by 100 μM of curine or guattegaumerine, suggesting that the two alkaloids could act as competitors of verapamil for binding onto P-gp.

#### 2.1.3. Determination of the Potential Drug Binding Sites through Docking Analysis

To investigate any potential interactions of curine, guattegaumerine, and verapamil with Mdr1b/P-gp proteins and to explain the inhibitory effect of these alkaloids, we performed docking analyses by using AutoDock 4.2.

To achieve this, we used the available LmrA PDB file (1MV5), which was used in the literature as a model for the P-gp protein because it can be a substitute for P-gp in human lung fibroblast MDR1 KO-cells [27]. In fact, P-gp in mammals is formed by a polypeptide chain of about 1280 amino acids that are symmetrically divided into an N-terminal region (MDL A) with a transmembrane domain (TMD1) associated with an intracytoplasmic loop with an ATP-binding domain (NBD1). The C-terminal region (MDL B) harbors the second transmembrane domain (TMD2) associated with the NBD2 domain. The efflux pump LmrA (access code: P97046) from *Lactococcus lactis*, a protein of 590 amino acids, consists of the association of two parallel peptide chains with two transmembrane domains forming a channel in the cytoplasmic membrane and two head-spade cytoplasmic NBD domains for ATP binding [28]. LmrA represents a good pattern for bacterial ABC transporters.

Openbabel was used to produce PDB files for all of the chemicals used in this study. Using AutoDock 4.2, we identified the potential interaction domains and binding sites for curine, guattegaumerine, and verapamil within rat Mdr1b and LmrA proteins. The positions occupied by curine and guattegaumerine on LmrA serve as models to explain the inhibitory effect observed on rhodamine efflux through P-gp in rat HTC-R cells. As an output of Autodock 4.2, inhibitory constants (Ki) were automatically estimated: 45 nM for curine, 137 nM for guattegaumerine, and 1.2 and 1.28 μM for verapamil.

**Figure 6:**
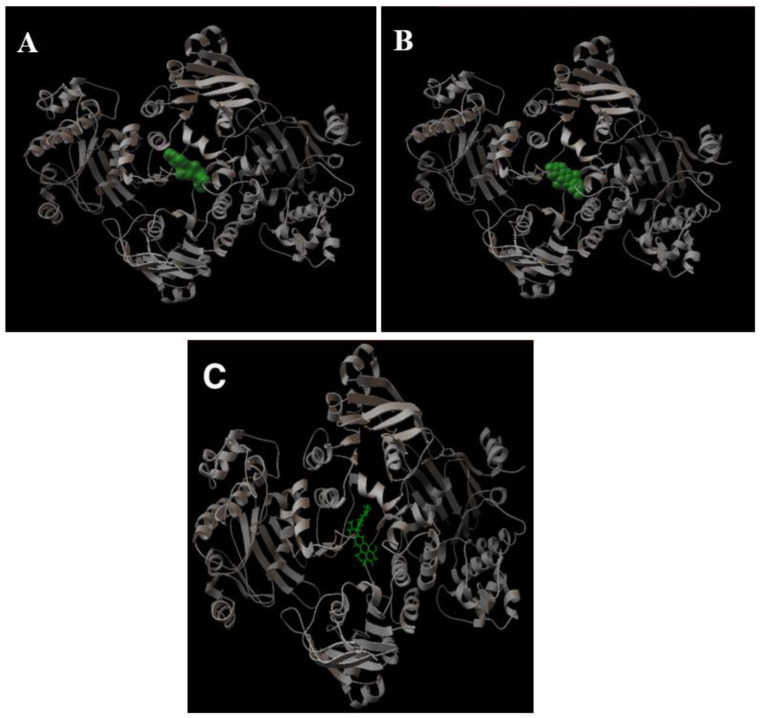
A 3D representation of the binding of verapamil, curine, and guattegaumerine on the LmrA protein. The pictures presented in the figure are a graphical output of AutoDock 4.2. All of the representations of LmrA (ribbon in gray) are in the same orientation. (**A**) Verapamil on LmrA; (**B**) curine on LmrA; (**C**) guattegaumerine on LmrA. Verapamil and curine are represented in terms of their volume (green) and guattegaumerine is shown in a stick representation.

Figure 6A shows a graphic representation of the binding of verapamil to LmrA. Verapamil interacts with the two chains and overflows within the cavity between the two NBDs at the bases of the transmembrane domains. Curine and guattegaumerine are also inhibitors of efflux through P-gp, and they appear to be competitors of verapamil. We looked for the region of the protein that interacts with drugs and the amino acids that were involved in this binding. Figure 6B shows the binding of curine with LmrA, and it can be noticed that the interaction domain of curine is very close to that of verapamil, whereas that of guattegaumerine is shifted (Figure 6C). Once positioned on LmrA, the molecules form a bridge between the two NBD domains and seem to occupy a volume that is more or less important in this cavity, depending on the degree of rotation between the two symmetrical parts of these molecules and the number of interaction points with the two NBD domains. This region of the molecule appears to form a pocket. The analysis of the 3D representation with RasMol confirms that the folding of the strand in this region delineates the contours of a pocket. Moreover, the binding domain of inhibitors used here is different from the rhodamine binding site. Indeed, Loo and Clarke [22] showed through the creation of P-gp mutants that rhodamine interacts with aliphatic amino acids belonging to transmembrane α-helices forming the outlet channel.

**Figure 7:**
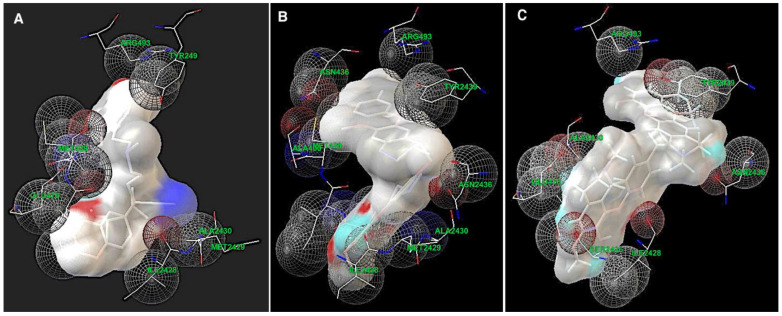
Areas of interaction between the amino acids of LmrA and the drugs. Alkaloids are represented in the volume and the amino acids of LmrA developing interactions with the drugs are represented in sticks. The spheres delineate domains of interaction. Amino acids are identified by their three-letter codes and their position numbers in the chains, with an additional number 2 for the second strand. (**A**) Verapamil; (**B**) curine; (**C**) guattegaumerine.

Using AutoDock and the auxiliary program AutoGrid, amino acids involved in the interactions were identified. Thus, in the LmrA model, verapamil, curine, and guattegaumerine interact with the two peptide chains forming the LmrA channel at the same amino acid sequences in the region between amino acids 426 and 493 in the NBD’s domain (Figure 7). These interactions are mostly of the Van der Waals type—hydrophobic interactions.

Figure 7 shows the amino acids involved in the interaction with the drugs in greater detail. Verapamil develops interactions with amino acids Ile428-Met429-Ala430 in the two NBD domains and with Tyr439, Gly478, and Arg493 on NBD1 (Figure 7A). Curine interacts with Ile428-Met429-Ala430 and Asn436 in the two NBD domains, as well as Arg493 in NBD1 and Tyr(2)-439 in NBD2 (Figure 7B). Guattegaumerine (Figure 7C) can also interact with LmrA in the same region, developing links with Ile428, Ala430, and Arg493 on NBD1 and Ser(2)-426, Ile(2)-428, Asn(2)-436, and Tyr(2)-439 on NBD2. It occupies the site in the highest position compared to the positioning of curine and verapamil.

We observed that there is a strong competition for the occupancy of sites between verapamil and curine and that the dissociation constant (represented by Ki) of curine is 30 times lower than that of verapamil. This could explain the removal of inhibition observed in the efflux experiments. Curine binds faster than verapamil on P-gp, preventing it from attaching and playing its role as an inhibitor of rhodamine efflux. However, curine is not exactly at the same position as verapamil in the binding site of P-gp and is more inclined to interact with NBD2 at Tyr (2)-439; thus, it does not fully operate on the pump, since there is only 41% inhibition.

Therefore, guattegaumerine is a weaker inhibitor of the pump (22%), but is still capable, with a Kd of 137 nM, which is 10 times lower than that of verapamil, of being a competitor of this molecule for its binding on P-gp.

All ABC transporters present the same organization of domains all along the polypeptide chain. The first transmembrane domain composed of at least four helices is linked to the first NBD domain by two cytoplasmic loops. The NBD is divided into several highly conserved sub-domains: the WalkerA/P-loop, Q-loop, ABC signature, Walker B, D-loop, and H-loop.

**Figure 8:**
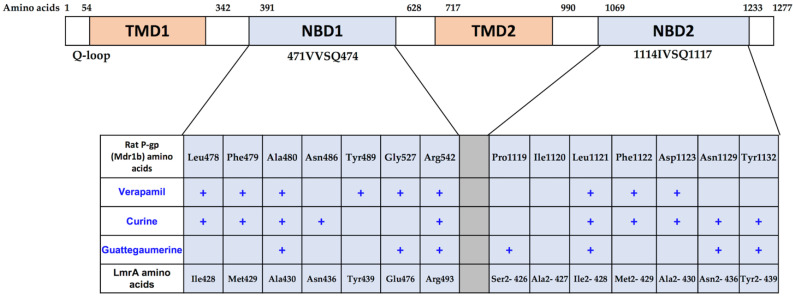
Diagram of rat Mdr1b and LmrA amino acids of the NDB domains involved in binding with drugs. The top of the figure shows a simple diagram of the protein with 1277 amino acids with the positions occupied by the two transmembrane domains (TMDs) and the two nucleotide-binding domains (NBD). The amino acids involved in drug binding deduced from the docking analysis of rat P-gp (Mdrlb) and LmrA are shown in the table. Amino acid sequences of rat Mdr1b and of *Lactococcus lactis* LmrA were obtained from the Swiss Prot database (access code: P43245) and UniProtKB/Swiss-Prot database (access code: P97046), respectively.

As shown in Figure 8, the number and the position of amino acids implicated in the binding with drugs can explain the efficiency of competition between these molecules. There is strong competition for the occupation of sites between verapamil and curine and, as the dissociation constant (represented by Ki) of curine is 30 times lower than that of verapamil, it could explain the removal of verapamil inhibition observed in the efflux experiments. Curine interacts faster than verapamil on Mdr1b(P-gp), preventing it from binding. However, curine is not exactly in the same position as verapamil on the site and is more inclined to interact with the NBD2 at Tyr1132; thus, it does not fully operate the pump because there is only 41% inhibition.

**Scheme 1:**
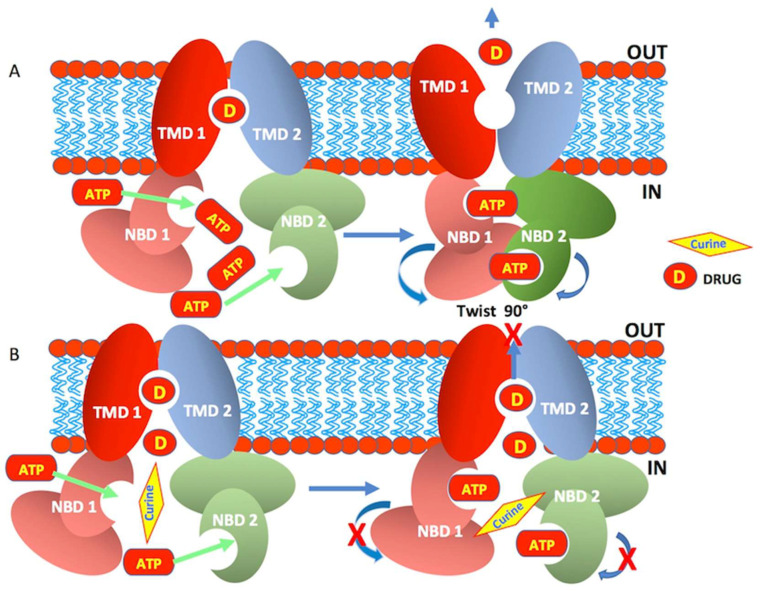
A hypothetical model that explains how curine affects the efflux activity of ABCB1. (**A**) displays a schematical representation of the ABCB1 membrane protein in the process of the efflux drug. (**A**) shows the transition from an inactive state to an active state of the efflux pump. The exclusion of a drug (D) through the channel, which is formed by the interaction of the two transmembrane domains (TMD1 and 2), occurs following the hydrolysis of ATP after its binding to the NBD1 and 2 sites. The energy released by this hydrolysis promotes a 90° rotation of the NBDs, thus allowing the opening of the channel and the efflux of the drug (D). (**B**) shows the inhibition of the efflux of the drug (D) following the binding of curine to the NBDs, thus preventing their rotation and the opening of the pump channel.

Figure 8 also shows the amino acids’ correspondence between rat Mdr1b and LmrA. From the docking analysis, amino acids potentially implicated in verapamil, curine, or guattegaumerine binding on LmrA were localized at three amino acids downstream from the Q-loop (Q424). Interestingly, the amino acid sequence VSQ inside the Q-loop was highly conserved in ABC transporters, with the glutamine (Q) in position 475 in NBD1 and in position 1118 in NBD2 of rat Mdr1. However, genetic evolution introduced several variations in the amino acid sequences in the regions surrounding the Q-loop, and the Ile-Met-Ala binding sequences for verapamil and curine in LmrA became Leu-Phe-Ala and Leu-Phe-Asp in NBD1 and NBD2 of Mdr1b, respectively, without a great change in hydrophobic properties. Scheme 1 represents our hypothetical model showing the mechanism of curine efflux by ABCB1.

## 3. Discussion

For the first time, we purified two bisbenzylisoquinolines, curine and guattegaumerine, from the roots of *Isolona hexaloba*. Physical and spectral analyses were used to determine the structure of curine (A). Meanwhile, for guattegaumerine (B), the resolution could only be achieved after hemi-synthesis of the triacetylated derivative form (C). 

These molecules had been previously tested for their therapeutic potential, with essentially antiparasitic, antiplasmodial, and antimitotic activities [6,7,13,19], as a vasodilator compound, as an inhibitor of calcium pumps in the case of curine [15,16], and in the induction of HepG2 cell death and cytokine secretion inhibition [17,18]. Concerning this latter property, we investigated the potential effect of curine and guattegaumerine on the efflux pumps responsible for cancer cell resistance to drugs.

It is well established that rhodamine is expelled from anticancer-drug-resistant cells by the plasma membrane P-glycoprotein (ABCB1) [21]. The kinetic analysis of rhodamine efflux by the HTC-R cells showed that curine and guattegaumerine probably act as inhibitors of the Mdr1b pump (abcb1). A comparison with verapamil, a known inhibitor of P-gp function [23], showed that curine and guattegaumerine were less effective than verapamil at the same concentration. The clear additive effect of curine and guattegaumerine suggests that these two closely related alkaloids act on two related but different targets on P-gp. As shown by the docking analysis, the open molecule guattegaumerine binds the ABC transporter less strongly than curine and interacts with the protein in a different manner (Figure 6C, Figure 7C and Figure 8). However, curine and guattegaumerine appeared to be good competitors of verapamil. Although these two alkaloids probably interact with different parts of the verapamil binding site on Mdr1b, the observed competition could be explained by structural similarities in certain parts of these molecules, particularly between the methylated aromatic cycles and the spatial arrangement of the planes containing the aromatic rings (Figure 1).

The in silico docking analysis of our molecules with the protein LmrA from *Lactococcus lactis* used as a model [28,29] also shows that curine and guattegaumerine interact with an amino acid sequence located downstream of the Q-loop in the nucleotide-binding domain (NBD) of the protein. This region of the protein is important for the functioning of the carrier. Indeed, according to the models described in the literature and the analysis of the data carried out by Becker et al. [30], the Q-loop amino acids should be involved in both nucleotide binding and formation of the NBD–TMD interface. In the Mdr1b protein, the glutamines 475 and 1118 of NBD1 and NBD2, respectively, which belong to the Q-loop, will interact with the γ-phosphate of the ATP molecules attached to the A-loop. Among the amino acids downstream of the Q-loop, phenylalanine 479 in NBD1 plays a key role in the development of hydrophobic interaction with valine 907, which is located in the middle of the cytoplasmic loop connecting TMD2 to NBD2. Phenylalanine 1122 would develop the same type of interaction with the cytoplasmic loop linking NBD1 to TMD1. The two NBDs would meet head to spade when they interact as a result of the binding of the ATPs on the two NBDs. To facilitate the dynamic opening of the transmembrane channel for substrate release, twisting must be involved (Scheme 1) [31,32,33]. The binding of alkaloids to phenylalanines 479 and 1122 downstream of glutamine 475 is expected to disrupt the formation of NBD interactions and cause the inhibition of drug transport. These theoretical results could explain the observed inhibitory effect on P-gp and the competition with verapamil for protein binding.

## 4. Materials and Methods

### 4.1. Plant Material

Roots of *Isolona hexaloba* Engl. and Diels (Annonaceae) were harvested in the locality of Sibang, near Libreville, east of Gabon, in February 2003. A voucher sample (#2082) was deposited at the National Herbarium of Gabon (Libreville). Samples were dried and powdered in the shade at room temperature.

### 4.2. Chemicals

All chemicals used in this study were of research-grade: verapamil, rhodamine 123, and neutral red were from Sigma Aldrich Chimie S.a.r.l (St. Quentin Fallavier, France), and crystal violet was from Merck Life Science BV (Hoeilaart, Belgium).

### 4.3. Alkaloidal Extraction and Isolation of the Bisbenzylisoquinolines

For the isolation of alkaloids, 225 g of root powder was submitted to a procedure aimed to isolate alkaloids by using solvent extraction and column and thin-layer chromatography. Briefly, the plant material was soaked with NH_4_OH (28%) and extracted several times with CH_2_Cl_2_. The organic fraction was extracted three times with HCl (1 N). The aqueous fraction was basified with NH_4_OH to pH 9 and was extracted with CH_2_Cl_2_ again. The CH_2_Cl_2_ extract was washed with H_2_O and dried over anhydrous sodium sulfate, and the solvent evaporated to afford the total tertiary alkaloid extract (AE) (485 mg). The AE was subjected to column chromatography over silica gel and eluted with CH_2_Cl_2_/CH_3_OH/(NH_4_OH, 28%): 90/10/0.5: *v/v/v* to afford 65 fractions. The fractions 14–29 (120 mg) and 32–33 (212 mg) were subjected to column chromatography again and gave the bisbenzylisoquinoline alkaloids curine (A) (8.5%) and guattegaumerine (B) (24.5%), respectively.

### 4.4. Triacetylguattegaumerine (C) Preparation

To a solution of guattegaumerine (30 mg, 0.05 mmol) in anhydrous pyridine (0.5 mL) was added an excess of acetic anhydride (0.5 mL, 5 mmol). The reaction mixture was stirred at room temperature for 24 h and then diluted with water and extracted with CH_2_Cl_2_; and the organic layer was dried over Na_2_SO_4_ before concentration under vacuum to yield the title compound as a brown solid (33 mg, 91%).

### 4.5. Molecular Structure Determination

The structure of isolated compounds was determined by physical and spectral data analysis: MS, ^1^H-NMR, ^13^C-NMR, HSQC, HMBC, and COSY. The structure of guattegaumerine was actually confirmed owing to the synthesis and spectral analysis of triacetylguattegaumerine (3). These structures (Figure 1) were in agreement with those published in the literature [34,35].

Curine (A)

^1^H NMR (500 MHz, CDCl_3_) δ 7.15 (1H, d, 8.4 Hz), 6.97 (1H, d, 8.2 Hz), 6.85 (1H, d, 7.8 Hz), 6,72 (1H, s), 6.70 (1H, d, 8.3 Hz), 6.68 (1H, m), 6.66 (1H, s), 6.59 (1H, s), 6.50 (1H, d, 7.8 Hz), 5,98 (1H; s), 5.28 (2H, large), 3.94 (3H, s), 3.93 (3H, s), 3,52 (1H, m), 3.51 (1H, m), 3.2 (2H, m), 2.90 (2H, m), 2,80 (2H, m), 2.81(2H, m), 2,60 (2H; m) 2.52 (3H, s), 2,40 (2H, m), 2.30 (3H, s).

^13^C NMR (125 MHz, CDCl_3_) δ 155.8, 149.2, 146.9, 146.8, 144.5, 143.8, 138.9, 137.7, 134.3, 134.2, 132.7, 130.1, 127.2, 125.7, 125.5, 124.8, 124.5, 121.3, 120.5, 116.0, 115.7, 113.7, 112.7, 108.5, 65.9, 60.9, 56.5, 56.4, 46.0, 44.3, 42.4, 42.2, 40.3, 40.1, 25.5, 22.3. HRMS (EI) calculated for C_36_H_38_O_6_N_2_ (M^+^) 594.$\;{\left[\alpha \right]}_{D}^{20}$: −298° (EtOH, c 0.8 mg/mL).

Guattegaumerine (B)

^1^H NMR (500 MHz, CDCl_3_) δ 7.02 (1H, dd, 8.6; 2.0 Hz), 7,01(1H, dd, 8.6; 2.0 Hz), 6.86 (1H, d, 8.6 Hz), 6.83 (1H, dd; 8.2, 2.0 Hz), 6,82 (1H, d, 8.6 Hz), 6.75 (1H, d, 8.2 Hz), 6.62 (1H, d, 2.0 Hz), 6.53 (1H, s), 6.48 (1H, s), 6,27 (1H, s), 6.21 (1H, s), 5.40 (3H, br.) 3.82 (3H, s), 3.81 (3H, s), 3.71 (1H, m), 3.62 (1H, m), 3.22 (1H, m), 3.12 (1H, m), 3.11 (1H, m), 3.10 (1H, m), 2.85 (1H, m), 2.83 (1H, m), 2.80 (1H, m), 2.76 (1H, m), 2.75 (1H, m); 2.71 (1H, m), 2.60 (1H, m), 2.54 (1H, m), 2.53 (3H, s), 2.48 (3H, s).

^13^C NMR (125MHz, CDCl_3_) δ 155.1, 145.7, 145.5, 145.3, 143.5, 143.4, 143.3, 134.3, 131.6, 130.7, 130.6, 129.2, 129.1, 125.3, 124.8, 124.7, 120.0, 117.8, 117.7, 115.9, 114.3, 114.0, 110.6, 110.5, 64.5, 64.4, 55.7, 55.6, 46.9, 46.4, 42.2, 42.1, 40.4, 40.3, 24.9, 24.7. HRMS (EI) calculated for C_36_H_40_O_6_N_2_ (M^+^) 596. ${\left[\alpha \right]}_{D}^{20}$: −29 (EtOH, c 0.8 mg/mL).

Triacetylguattegaumerine (C)

^1^H NMR (500 MHz, CDCl_3_) δ 7.08 (1H, dd, 8.6; 2.0 Hz), 7,07(1H, dd, 8.6; 2.0 Hz), 6.89 (1H, d, 8.6 Hz), 6.85 (1H, dd; 8.2, 2.0 Hz), 6,84 (1H, d, 8.6 Hz), 6.83 (1H, d, 8.2 Hz), 6.80 (1H, d, 2.0 Hz), 6.66 (1H, s), 6.63 (1H, s), 6,50 (1H, s), 6.42 (1H, s), 3.84 (3H, s), 3.82 (3H, s), 3.72 (1H, m), 3.63 (1H, m), 3.25 (1H, m), 3.15 (1H, m), 3.12 (1H, m), 3.08 (1H, m), 2.86 (1H, m), 2.84(1H, m), 2.80 (1H, m), 2.77 (1H, m), 2.76 (1H, m); 2.71 (1H, m), 2.60 (1H, m), 2.54 (1H, m), 2.51 (3H, s), 2.45 (3H, s), 2.28 (3H, s), 2.27 (3H, s), 2.15 (3H, s).

^13^C NMR (125 MHz, CDCl_3_) d 169.0, 168.9, 168.7, 155.3, 149.3, 149.2, 147.7, 139.8, 138.4, 134.1, 132.7, 132.4, 131.0, 130.6, 130.5, 129.1, 129.2, 125.1, 123.0, 121.9, 121.8., 121.7, 121.4, 117.8, 117.7, 112.3, 112.2, 64.4, 63.9, 55.7, 55.6, 46.8, 46.7, 42.5, 42.4, 40.7, 40.6, 25.5, 25.4, 20.5, 20.4, 20.3. HRMS (EI) calculated for C_42_H_46_O_9_N_2_ (M^+^) 722.

The spectral data of the isolated compounds are available in the Supplementary Materials.

### 4.6. Cell Line and Culture Medium

The HTC-R cell line was developed from rat hepatoma carcinoma cells (HTC) through exposure to increasing concentrations of glycocholic acid ester (GCE), which induced resistance to biliary acids by activating Mdr gene expression. These cells constitutively expressed the P-glycoprotein (P-gp) efflux pump and became resistant to several anticancer drugs [26].

Cells were grown in Dulbecco’s Modified Eagle Medium (DMEM), supplemented with 10% fetal calf serum (FCS), 2 mM glutamine, 50 μg/mL streptomycin, and 50 units/mL penicillin.

### 4.7. Cellular Toxicity of Curine and Guattegaumerine

HTC-R cells were grown in 24-well plates in the complemented DMEM medium (1 mL per well) at 37 °C in a stove with an atmosphere of 5% carbon dioxide. When cells were confluent, the medium was removed, a complete medium containing various concentrations of curine or guattegaumerine was added, and the incubation was continued for 24 h. Then, the medium was withdrawn; the cells were gently washed with PBS buffer, and then set for 10 min with methanol.

The cells were then stained with 0.1% crystal violet for 15 min at room temperature. After incubation, the dye was removed, the cells were washed 3 times with PBS buffer, and then the dye remaining in the cells was extracted with 1% SDS for 20 min at room temperature. The absorbance at 595 nm measured in each well containing curine or guattegaumerine was reported in comparison to the absorbance measured in the control wells without the drug. The experiments were carried out in duplicate on 3 independent cell cultures.

### 4.8. Effects of Curine and Guattegaumerine on Cell Proliferation

HTC-R cells were grown in 24-well plates in a complete DMEM medium (1 mL/well). At t0, 1 × 10^5^ cells were placed in each well with or without increasing concentrations of curine or guattegaumerine between 168 µM and 16.8 nM. After 36 h of incubation at 37 °C, the culture medium was removed and the cells were gently washed 2 times with PBS buffer preheated to 37 °C. A total of 1 mL of a solution of 1% neutral red was added to each well, and the plates were incubated further for 10 min at 37 °C. Then, the dye was removed, the cells were washed twice with PBS, and 500 μL of methanol was added to each well to fix the cells and solubilize the dye. Absorbance at 460 nm was then measured, and values in wells containing drugs were compared to the values of absorbance in control wells. Experiments were carried out in duplicate on 3 independent cell cultures.

### 4.9. Effects of Curine, Guattegaumerine, and Triacetylguattegaumerine on Efflux Mediated by the P-gp Pump

In normal cells, rhodamine, a lipophilic fluorescent molecule, accumulates in the mitochondria. In cells overexpressing P-gp, rhodamine accumulates in the mitochondria, and especially in the cytosol. Then, efflux of rhodamine takes place through the P-gp protein. HTC-R cells were grown in 6-well plates up to confluence and in a DMEM medium containing GCE to maintain the overexpression of the Mdr1 gene. The cells were then washed twice with PBS buffer preheated to 37 °C and once with DMEM. The accumulation of rhodamine was carried out by cells for 90 min at 37 °C in complete DMEM containing 10 μM rhodamine 123.

After incubation, the medium was removed and cells were gently washed 6 times with PBS at 4 °C to block all biological efflux systems. The kinetics of efflux was initiated by the addition of PBS at 37 °C containing 100 μM verapamil or not, or 100 μM curine or guattegaumerine. At t + 1, 5, 10, 20, 30, and 60 min, the culture medium was taken from each well, kept in the ice, and replaced by preheated PBS containing the same molecules at the same concentrations. After the last sample was discarded, cells were lysed by adding 1 mL of Tris-HCl buffer at 50 mM and pH 7.5 containing 2% SDS. The lysate was recovered and centrifuged, and the supernatant was recovered to measure the amount of intracellular rhodamine at the end of the experiment.

The fluorescence of rhodamine in each sample was measured in a Perkin–Elmer spectrofluorometer. Rhodamine was excited at a wavelength of 509 nm, and the maximum emission wavelength of fluorescence was 529 nm. The sum of fluorescence of each sample in one experiment gave the total quantity of rhodamine accumulated by the cells. The value of each sample was then reported in comparison to the total value to estimate the percentage of efflux of rhodamine and to plot the kinetics. The initial rate for each kinetic was calculated from the slope at the origin of the straight line.

### 4.10. Docking Method

The structures were prepared for the docking study as follows: For the protein, waters were removed from the PDB file and hydrogen atoms were added; Gasteiger charges, atomic solvation parameters, and fragmental volumes were merged to the receptor. For all ligands, structures were built using the 2D editor Chemsketch. The 3D structures were generated using OpenBabel 2.2.0 and were minimized to a local energy minimum using the CHARMm-like force field. Gasteiger charges were assigned and non-polar hydrogen atoms were merged using AutodockTools. All torsions were allowed to rotate during docking.

The docking energy grid was produced with the auxiliary program AutoGrid. The grid dimensions were 40 × 40 × 40 points along the x-, y- and z-axes, with points separated by 0.375 Å. The grids were chosen to be sufficiently large to cover the probable interaction pocket of ligands inside the channel. The center of the ligands was positioned at the grid center. The Lamarckian genetic algorithm was used, and the energy evaluations were set to default values. Other parameters were set to the default values.

## 5. Conclusions

Two bisbenzylisoquinolines (curine and guattegaumerine) were purified from an extract of the roots of *Isolona hexaloba* species. The structures of these molecules and the function of curine as an anti-vasodilator and inhibitor of calcium pumps led us to investigate whether these molecules have cytotoxic or antiproliferative activity. Our results show that, at a concentration of 168 μM, curine and guattegaumerine inhibit cell proliferation by 90% and 70%, respectively. For concentrations below 100 μM, curine and guattegaumerine show no cytotoxic or antiproliferative effects. However, these molecules can act specifically on the efflux pumps and are more particularly capable of inhibiting the release of rhodamine by P-gp in HTC-R cells that are multi-resistant to anticancer agents. Docking analysis of the binding of verapamil, curine, and guattegaumerine on LmrA, an ABC transporter similar to P-gp, shows that these molecules bind the transporter near the Q-loop, preventing the correct binding of ATP. Thus, the opening of the channel and the initiation of ATP hydrolysis, which allows drug efflux, were inhibited. Altogether, our results show that curine and guattegaumerine act on rat hepatocarcinoma cells by inhibiting the Mdr1b efflux pump. Since curine and guattegaumerine appear to have low cytotoxicity in confluent cells and a significant inhibitory effect on P-gp, these alkaloids open promising avenues for the development of new therapeutic agents in terms of both cancer and treatment of infectious diseases, as demonstrated by Hulen et al. [36]. Thus, these types of molecules that are weak inhibitors of P-gp could serve as precursors to chemical modifications that would lead to the design of active and efficient therapeutic molecules.

## Data Availability

Not applicable.

## Supplementary Materials

The following are available online at https://www.mdpi.com/article/10.3390/molecules27093030/s1.

## Sample Availability

Samples of the compounds are not available from the authors.
