# Supplementary material for: Effects of Two Natural Bisbenzylisoquinolines, Curine and Guattegaumerine, Extracted from Isolona hexaloba on Rhodamine Efflux by Abcb1b from Rat Glycocholic-Acid-Resistant Hepatocarcinoma Cells
Source: Molecules. 2022 May 9;27(9):3030. doi: 10.3390/molecules27093030 (PMC9099951; doi:10.3390/molecules27093030)

Supplementary data

Curine (A)

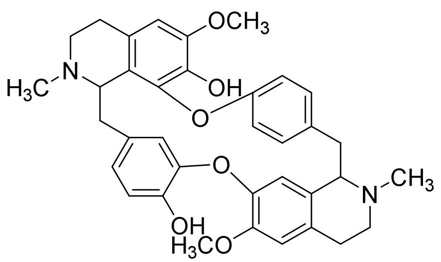

Curine - ^{13}C NMR (125 MHz, $CDCl_3$)

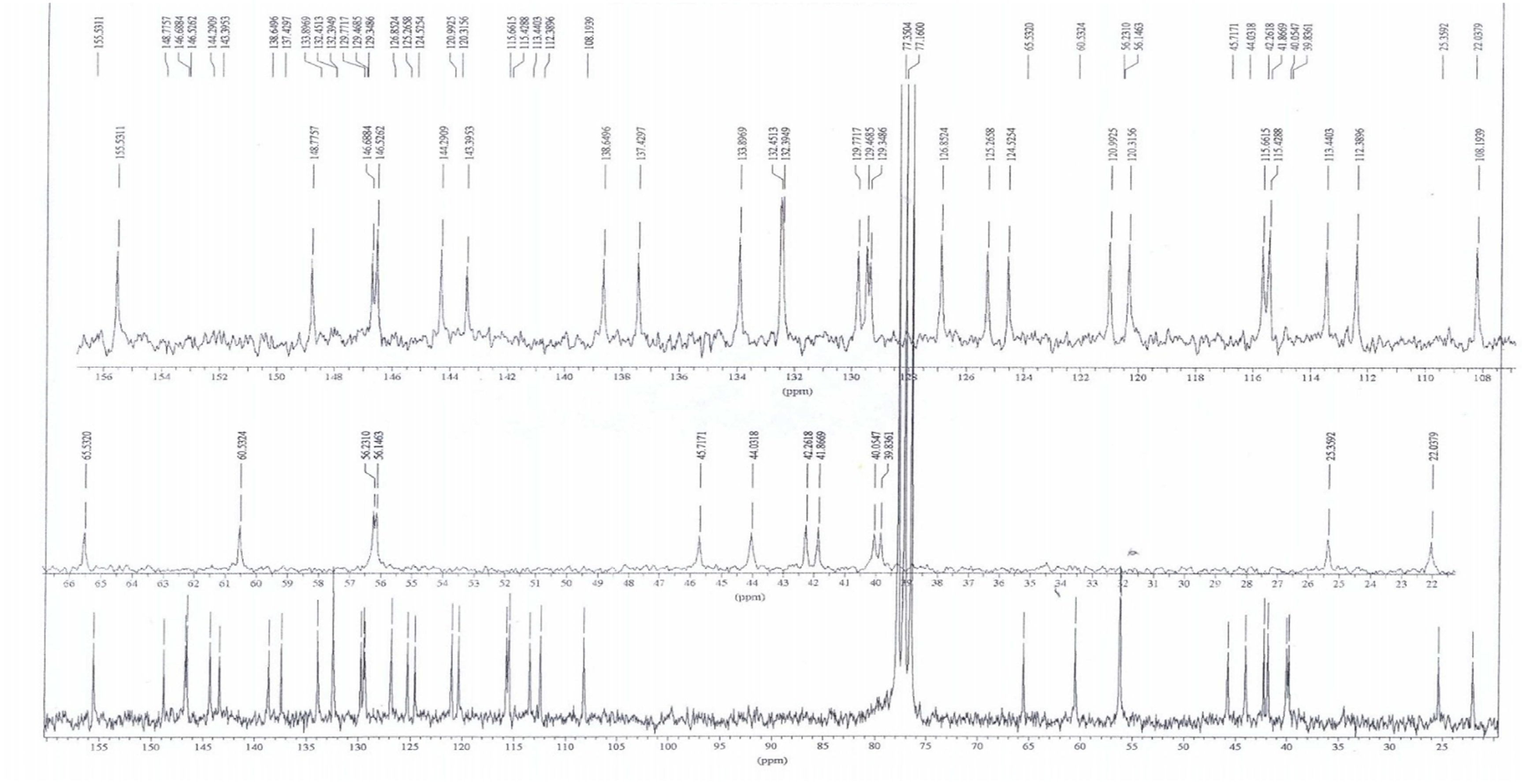

Curine - ^{13}C NMR (125 MHz, $CDCl_3$) and DEPT 135°

Curine **Zone (0 – 70 ppm)**

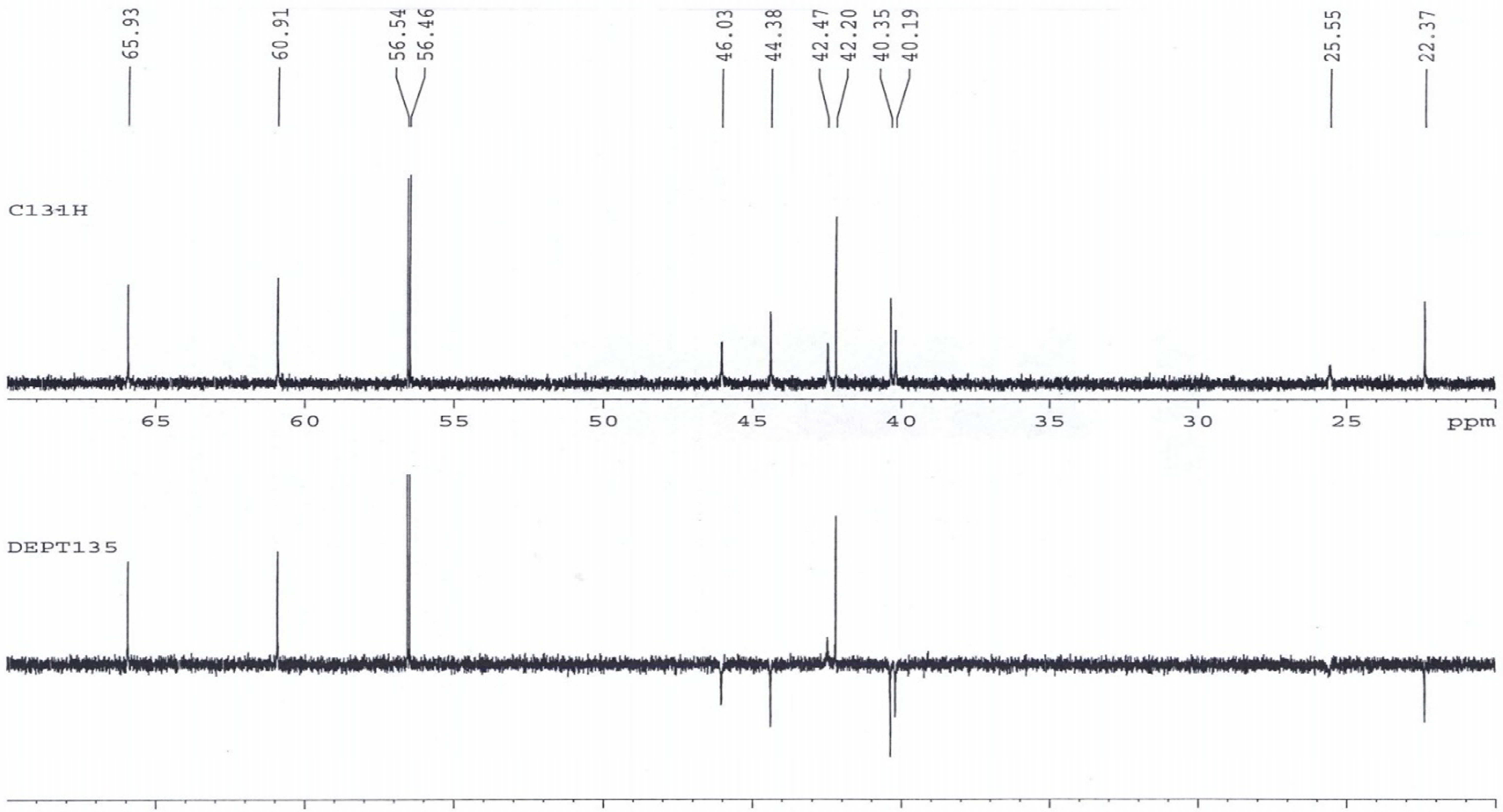

Curine **Zone (100-160 ppm)**

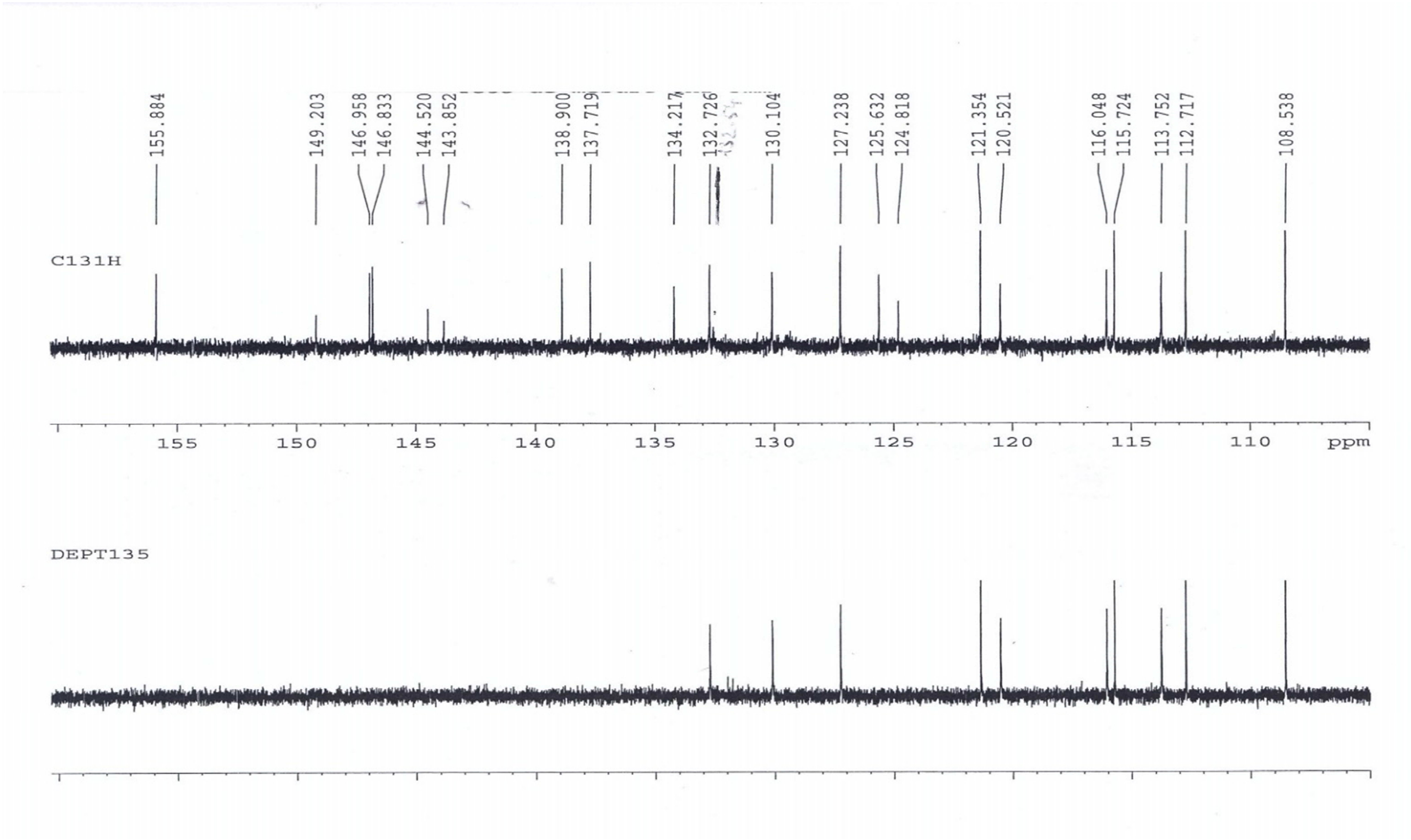

Curine - ^{13}C – ^{1}H correlations in HSQC NMR, $CDCl_3$

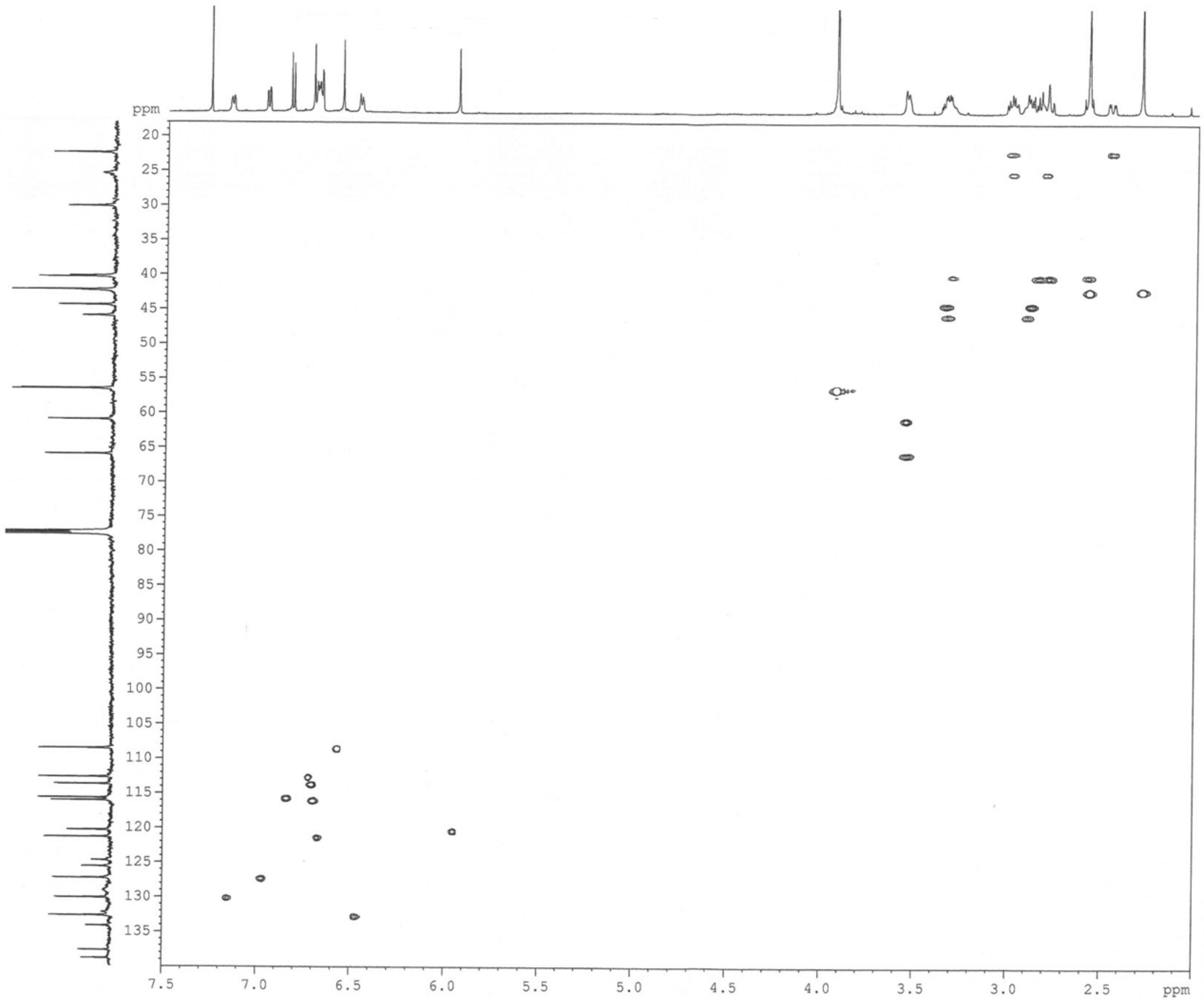

Guattegaumerine (B)

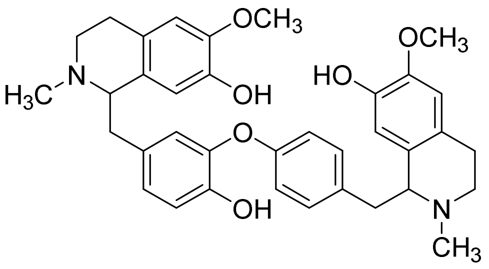

Guattegaumerine - ^{1}H NMR (500 MHz, $CDCl_3$)

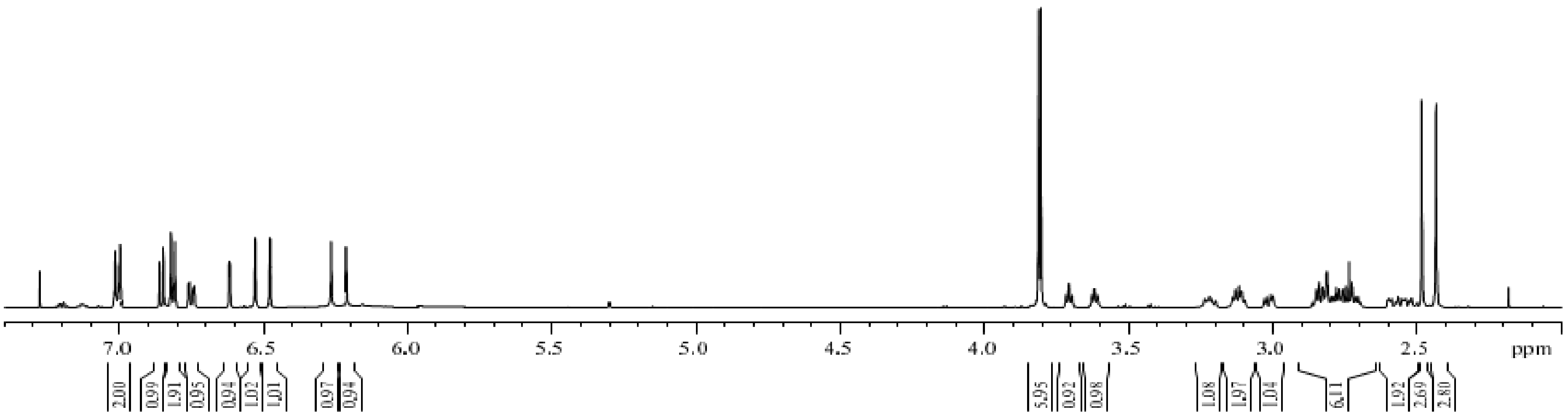

Guattegaumerine - ^{13}C NMR (125 MHz, $CDCl_3$)

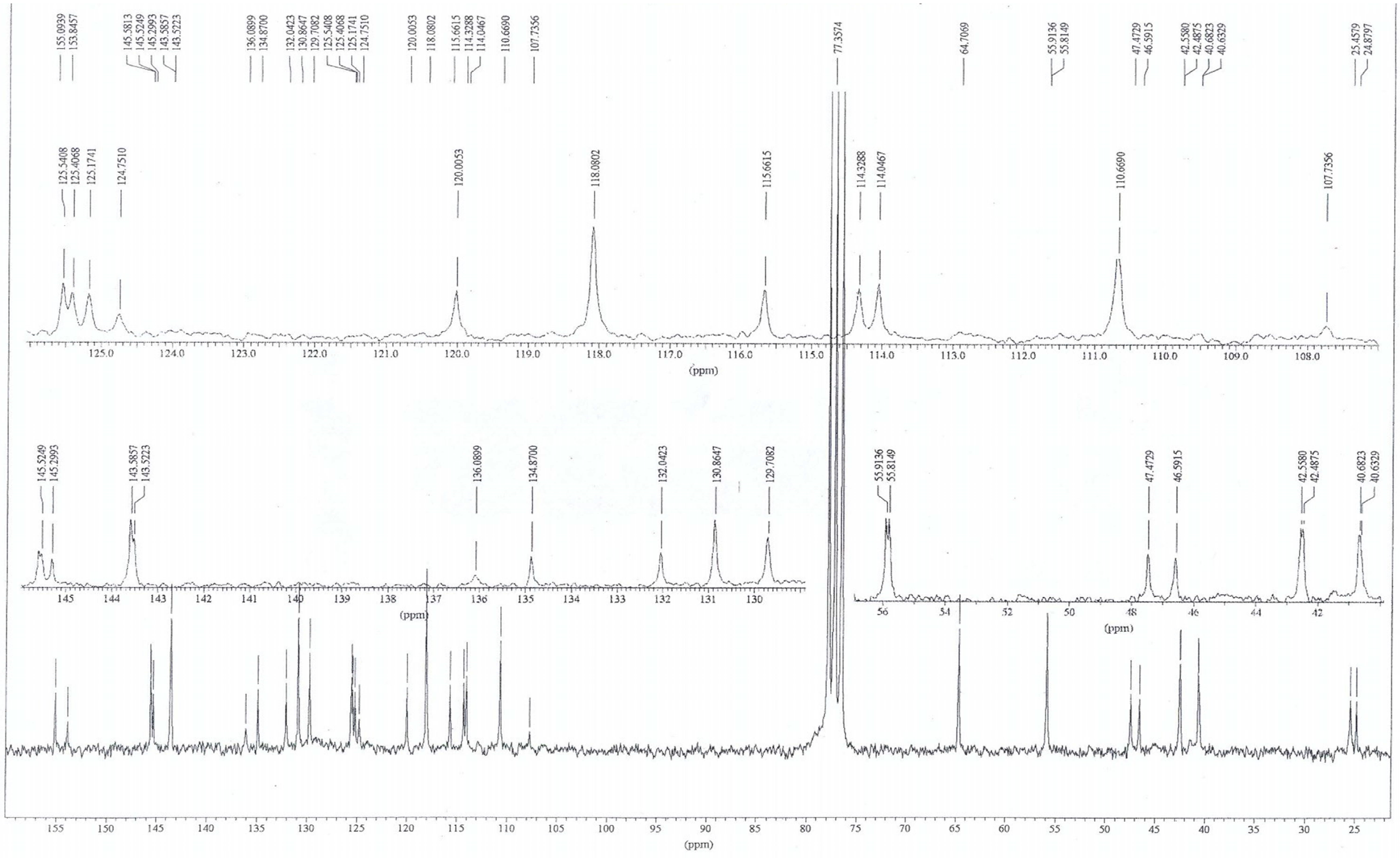

Triacetylguattegaumerine (C)

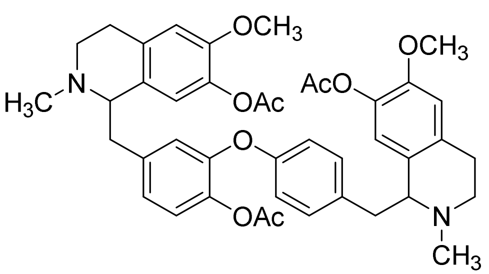

Triacetylguattegaumerine ^{1}H-NMR (500MHz, $CDCl_3$)

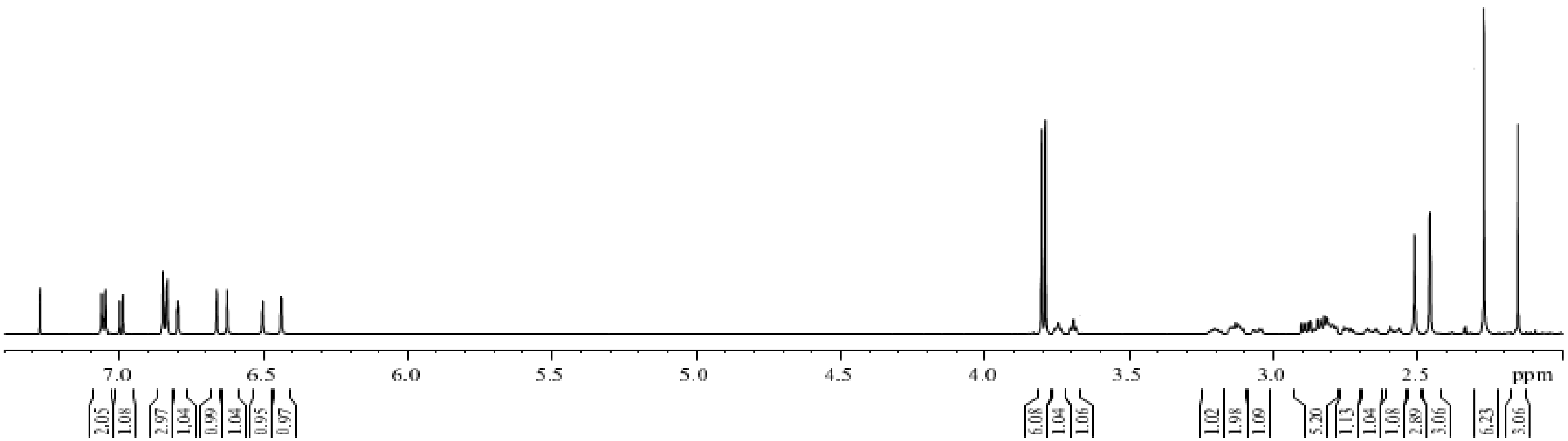

Triacetylguattegaumerine ^{13}C-NMR (125MHz, $CDCl_3$) and DEPT 135°

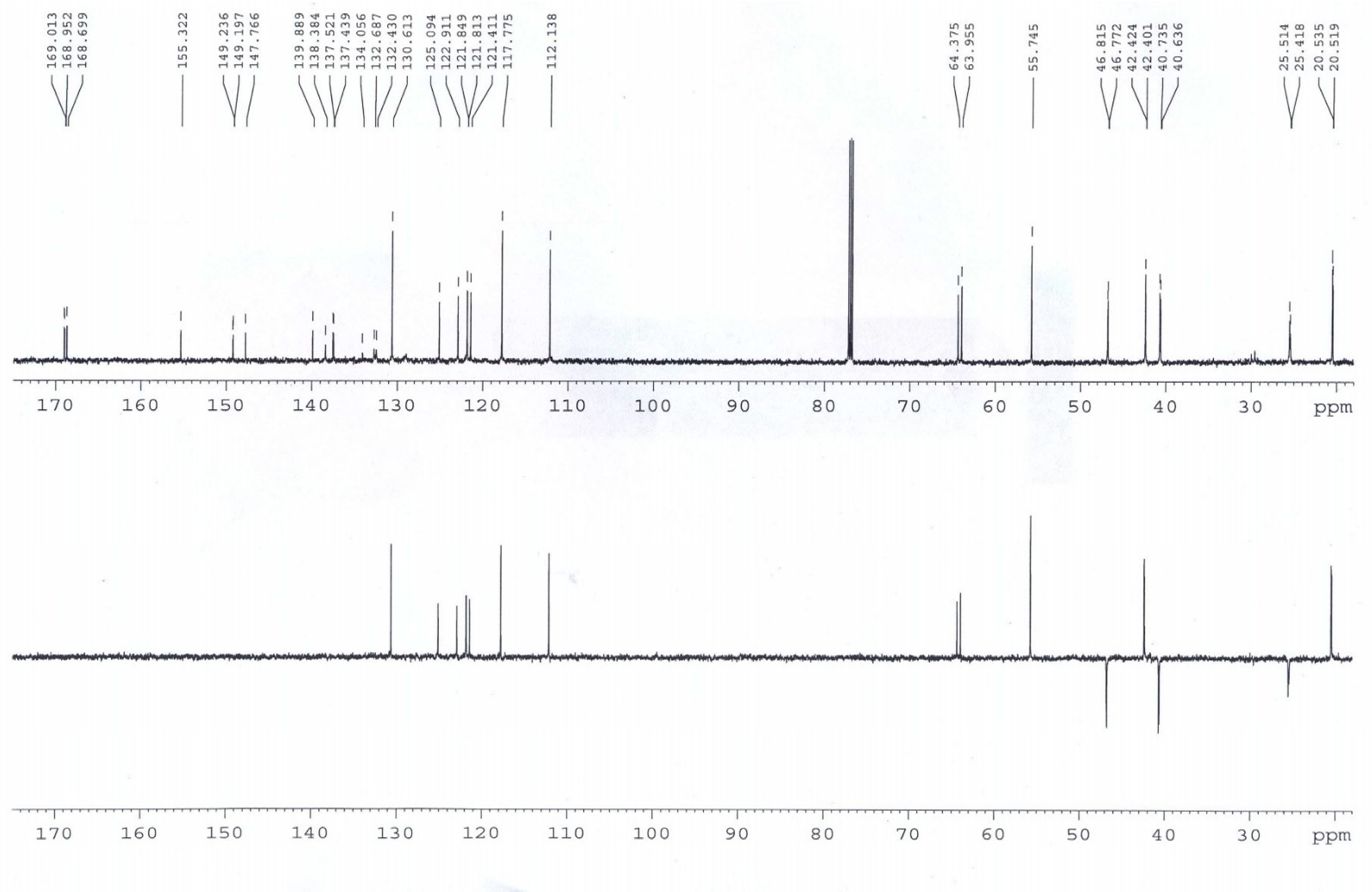

Supplement: Supplementary file 1 [file molecules-27-03030-s001.zip › molecules-1677286-supplementary.pdf]
